# The influence of contemporary and emerging factors on blood lead concentrations among young males in conflict with the law: a case study from a middle-income country

**DOI:** 10.3389/fepid.2024.1425604

**Published:** 2025-01-07

**Authors:** Thokozani P. Mbonane, Andre Swart, Angela Mathee, Nisha Naicker

**Affiliations:** ^1^ Department of Environmental Health, Faculty of Health Sciences, University of Johannesburg, Johannesburg, South Africa; ^2^ Environment and Health Research Unit, South African Medical Research Council, Johannesburg, South Africa; ^3^ Epidemiology and Surveillance Section, National Institute for Occupational Health, a Division of the National Health Laboratory Services, Johannesburg, South Africa

**Keywords:** blood lead concentration, contemporary factors, emerging factors, young males, conflict with the law, environmental exposure

## Abstract

**Introduction:**

Scientific evidence shows that contemporary and emerging factors contribute to high blood lead concentrations in different populations. The study aimed to determine blood lead concentrations and risk factors associated with high blood lead concentrations among young males in conflict with the law.

**Methods:**

A cross-sectional analytical study was conducted among 192 conveniently selected participants from two youth secure (correctional) facilities in Gauteng Province, South Africa.

**Results:**

The study's overall blood lead concentration median was 3.30 μg/dl, ranging from 0.85 to 48.11 μg/dl. Young males born outside of South Africa (median = 8.78 μg/dl) and in villages (median = 4.95 μg/dl), working before coming to the facility (median = 5.23 μg/dl) and involvement in illegal mining (median = 9.00 μg/dl) had high blood lead concentrations in this study. Contemporary and emerging risk factors such as being born outside the country (AOR: 3.10, 95%CI: 1.01–1.88), involvement in illegal mining activities (AOR: 1.36, 95%CI: 1.14–1.91) and staying in a house with peeling paint on the outside (AOR: 2.26, 95%CI: 1.12–4.30) were found to influence blood lead concentration.

**Discussion:**

The study findings show that contemporary (co-existing) and emerging factors influence blood lead concentrations. Therefore, there is a need to investigate these factors further in communities that may be affected. Lastly, there is a need for a holistic approach involving multiple sectors to introduce human lead concentration screening and preventive programmes.

## 1 Introduction

Lead-contaminated environments continue to be persistent and are often ignored environmental health hazards in low and middle-income countries (LMICs), especially in the Sub-Saharan African region (1). Most sources of lead contamination or exposure are linked to human activities and the most affected are the poorer communities within this region (2, 3). Contaminated lead environments have a direct impact on human blood lead concentration (4). Consequently, high and low blood lead concentrations contribute to the development of adverse health and behavioural issues (5, 6). Scientific evidence shows that contemporary (co-existing) factors are usually responsible for blood lead such as socio-economic, behavioural (habits and lifestyle) factors as well as occupational (7–11). In LMICs, these factors included metal water plumbing, cottage industries, residing near lead emitting industries such as mines and staying in old or dilapidated houses with peeling leaded paint, and lastly being a young male due to outdoor activities and being born in a country without lead prevention programmes (12–16).

African countries have experienced a surge of emerging lead sources that contribute to high blood lead concentrations which indirectly can lead to severe adverse health effects. Artisanal and illegal mining, making and using aluminium cookware (pots) are a few emerging lead sources in Africa (17–21). Numerous activities in illegal mining contribute to high blood lead concentrations such as the use of heavy metals in retrieving gold, and the lack of personal protective clothing mostly these heavy metals end up in their surrounding environment such as soil, water, and vegetation (22, 23). Similarly, aluminium cookware making where recycled scrap metals are melted in the making of these pots (17, 24, 25). Once the surrounding environment is lead-contaminated it poses a risk for high blood lead concentrations for people involved in these activities and surrounding communities (18, 24, 25). In Nigeria in 2010, the country experienced lead poisoning outbreaks due to the contaminated environment that led to the death of more than 100 children due to illegal gold mining (20). A recent case study in South Africa reported high blood lead concentrations linked to illegal mining activities among young males aged 18 and 15 years old with levels of 48.11 and 35.76 µg/dl respectively (19).

Studies conducted in high-income countries have reported high human lead concentration among populations in conflict with the law (26–30). In the United States of America (USA), researchers have established a connection between crime with lead exposure and severe lead poisoning, including violent and age-specific violent crimes within incarcerated populations (31–33). Therefore, it is crucial to ascertain the prevalence of lead poisoning among these populations in South Africa, a nation that is grappling with elevated rates of violent crime. Furthermore, there are no studies that have been conducted in LMICs on these populations. This is despite recent studies in South Africa showing a link between blood lead concentrations with violent and criminal behaviour among teenagers and young adults in the general population especially among young males (34–36). Hence current study assessed the blood lead concentrations among young males in conflict with the law. Furthermore, it determined the emerging and contemporary risk factors associated with high blood lead concentrations in the study population.

## 2 Methodology

### 2.1 Study design and setting

We conducted a cross-sectional analytical study among young males in conflict with the law in Gauteng Province, South Africa. Gauteng Province is one of the South African provinces with a high crime rate (37). Between 2005 and 2016, the province had the highest total crimes (estimated at around 600,000) reported among the nine provinces in South Africa (37). It also has the highest number of young people in the criminal justice system compared to most provinces in South Africa (37).

### 2.2 Study population

The targeted population was young males in conflict with the law. These young males were 19 years or younger when the study was conducted. In South Africa, children who are in conflict with the law (committed a crime) and sentenced are kept in secure facilities (places for rehabilitation for children younger than 18 years that are in conflict with the law). These facilities are managed by the Gauteng Department of Social Development to ensure the rehabilitation of young children in conflict with the law. The young males were housed in two secured facilities (namely A = capable of 200 capacity & B = capacity of 100), found in the City of Johannesburg and the City of Tshwane. A convenience sampling approach was used to reach and select participants in the study. The study sample size was determined using a population of 300 based on the total capacity of the two secure facilities at the time and using the prevalence of 30% of high blood lead concentrations among violent males (36) The following formula $n=1.96^{2}*(\;p*q)÷ME^{2}$ was used, where the *p*-value was 0.05, study power (*q*) was 80% and proportion difference (ME) was 5%. The estimated sample size for the large population is *n* = 323. However, the population was smaller than the required sample size. Therefore, the sample size was adjusted using the following equation: *n* (adj) = (N*n)/(*N* + *n*). Hence, the required sample for the study was set at 192.

The recruitment process involved approval from the authorities to approach secure facilities. Thereafter, a list of current young males in the facilities was acquired from the facility manager. We then approached the parents and guardians of young males than 18 years old to consent before asking the male juveniles to assent to participate. At the same time, young males over 18 years old were approached for consent to participate in the study, this process is described in detail elsewhere (38).

### 2.3 Sampling

To determine blood lead concentrations in the study population, a professional South African Nursing Council registered nurse was appointed for blood withdrawal. Venous blood (5–7 ml) was withdrawn from each participant using a sterile test tube (BD Vacutainer system) containing the anticoagulant ethylene diamine tetra-acetic acid (EDTA). Then, the blood samples were transported and submitted within 24 h of collection to an accredited laboratory in Johannesburg. At the laboratory, the flameless atomic absorption method (Model Perkin-Elmer Analyst 300 with HGA 850), adapted from Baily, was used to measure the lead concentrations in the blood. The limit of detection for blood lead was 0.1 μg/dl.

### 2.4 Socio-demographic and environmental factors

Socio-demographics (study participants' characteristics), and emerging and contemporary factors that influence blood lead concentrations were collected using an adapted questionnaire, previously used in other local studies (10, 34–36, 39–41). The questionnaire was translated from English to two local languages (isiZulu and Se-Sotho) and administered with the assistance of trained research assistants.

The first section of the questionnaire covered the socio-demographic information which included age categorized into three (15 years or younger/16–18 years/19 years), educational level (no schooling/primary school/high school), birth country (South Africa/Outside South Africa), local birthplace (township/town/informal settlement/village or rural), occupational status prior being in the facility (not working/working) and smoking status (no/yes).

The following environmental factors were collected in the second section of the questionnaire; living near a lead source (mine or mine tailing/lead emitting factories/non-emitting industries), house structure (formal/informal), plumbing (metal/plastic/no plumbing), exterior peeling paint (no/yes), interior peeling paint (no/yes), traditional medicine use (no/yes), cottage business where participants grew-up (no/yes) and household overcrowding (no/moderate/extreme overcrowding). Overcrowding status was determined by calculating the ratio of the number of occupants/people per bedroom. Therefore, based on Nkosi and colleagues and UN-Habitat, overcrowding was classified as “no overcrowding”-less than 2 occupants per bedroom, “moderate overcrowding”- where 2–5 per bedroom and “extreme overcrowding”- where more than 6 occupants per bedroom (42, 43).

### 2.5 Data management and analysis

**Figure 1:**
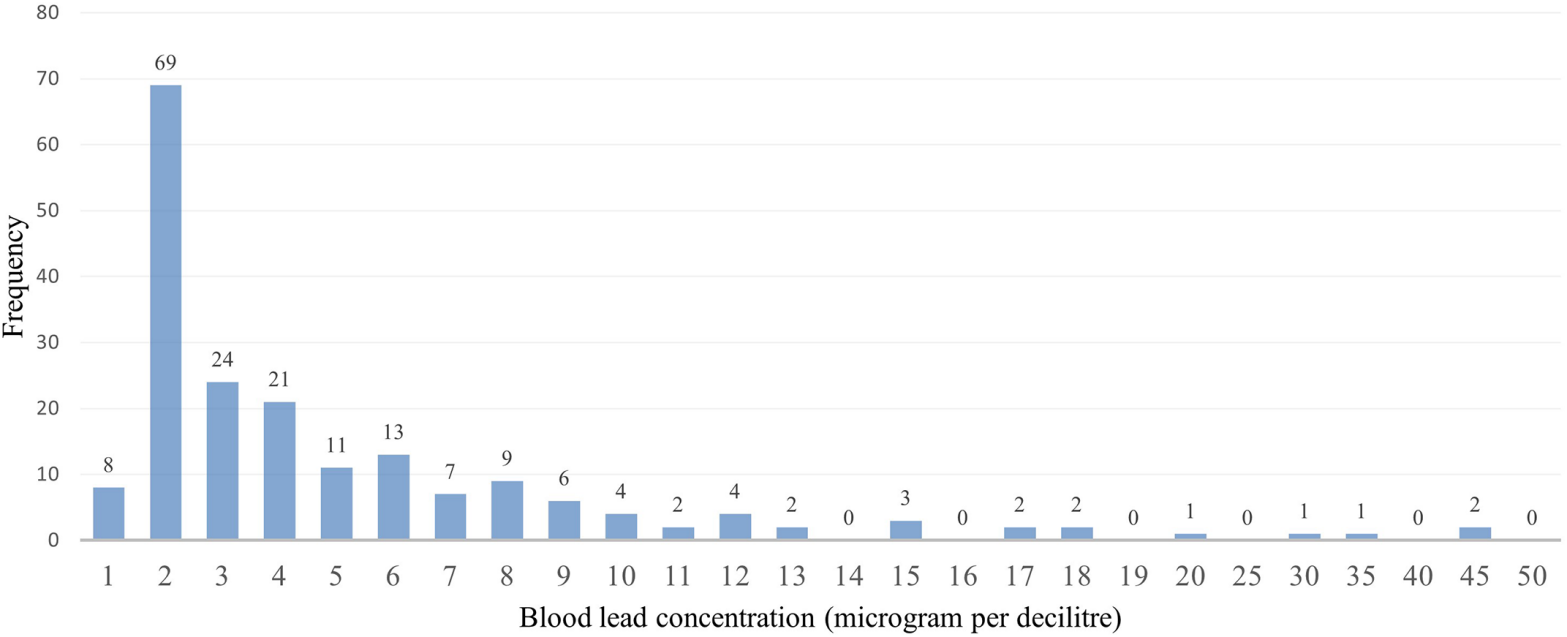
Abnormal blood lead concentration distribution.

**Figure 2:**
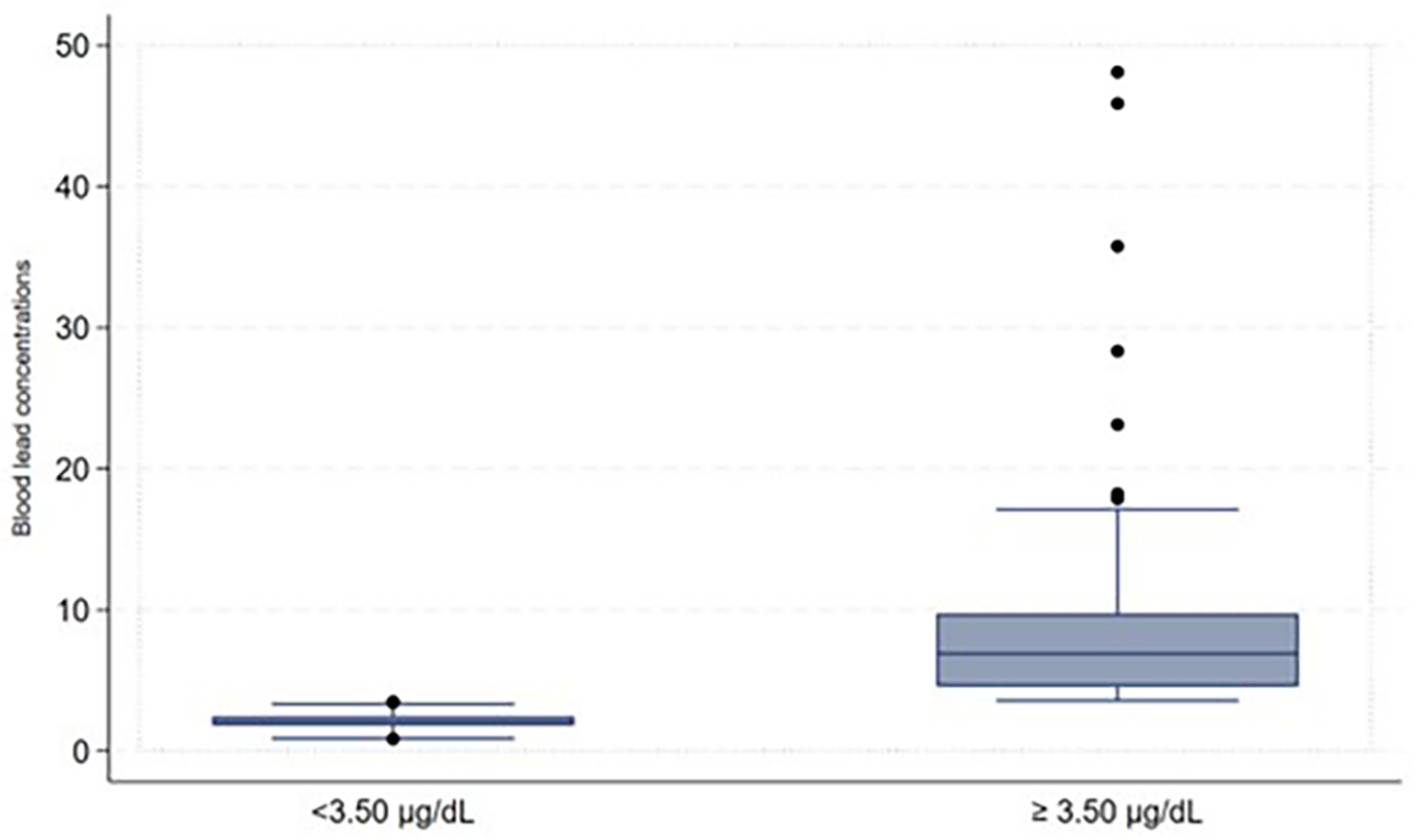
Blood lead concentration comparison.

Some participants indicated that they were working before coming to the facilities and were asked about the type of work they were involved in, we then created a variable to focus on those involved in illegal mining and those not involved in illegal mining. This variable was created because involvement in artisanal gold mining contributes to high blood lead concentrations (21). The captured data on the Redcap was exported to Microsoft Excel for cleaning, sorting, and coding before moving it to the STATA version 15.1 Statistics program for descriptive and inferential analysis purposes. The study population's demographic characteristics (categorical variables) were described using frequencies and proportions. The distribution of the blood lead levels as continuous variables underwent checking for normal distribution. Because of its skewed distribution, the blood lead concentrations as shown in Figure 1 were arranged and described as a categorical variable to display low and high blood lead concentrations (shown in Figure 2) in the study. Blood lead concentrations were dichotomized [into < 3.50 µg/dl (low) and ≥3.50 µg/dl (high)] based on the updated recommendation for blood lead reference value by CDC (44, 45). The majority of participants in the study had blood lead concentrations of <3.50 µg/dl representing low blood lead concentrations (*n* = 101; 53%) and 91 (47%) participants had blood lead concentrations of ≥3.50 µg/dl (representing high blood lead concentrations), as shown in Figure 2.

Binary logistic regression was used to determine and describe factors associated with blood lead concentrations. Where the dependent variable was blood lead concentrations bivariate analysis was used to determine an association between blood lead concentration with individual factors and was reported as a crude odds ratio (COR). A backward stepwise elimination method was used in the multivariate analysis [association reported using adjusted odds ratio (AOR)] to determine the factors associated with high blood lead concentrations. The unadjusted and adjusted odds ratios were reported with a 95% confidence interval (95%CI). To illustrate a statistically significant association the *p*-value was set at 0.05 in the bivariate and multivariate analysis.

### 2.6 Ethical considerations

The study was conducted according to the guidelines of the Declaration of Helsinki and approved by the Research Ethics Committee of the University of Johannesburg, Faculty of Health Science (REC-01-64-2018 and 27 June 2018) and South African Medical Research Council Human Research Ethics Committee (EC011-7/2019 and 28 January 2020). The study was granted permission by the Gauteng Department of Social Development (2/9/05). Lastly, informed and assent consent was obtained from all subjects involved in the study, including guardians of young males under 18 years old.

## 3 Methodology

### 3.1 Blood lead concentrations

**Figure 3:**
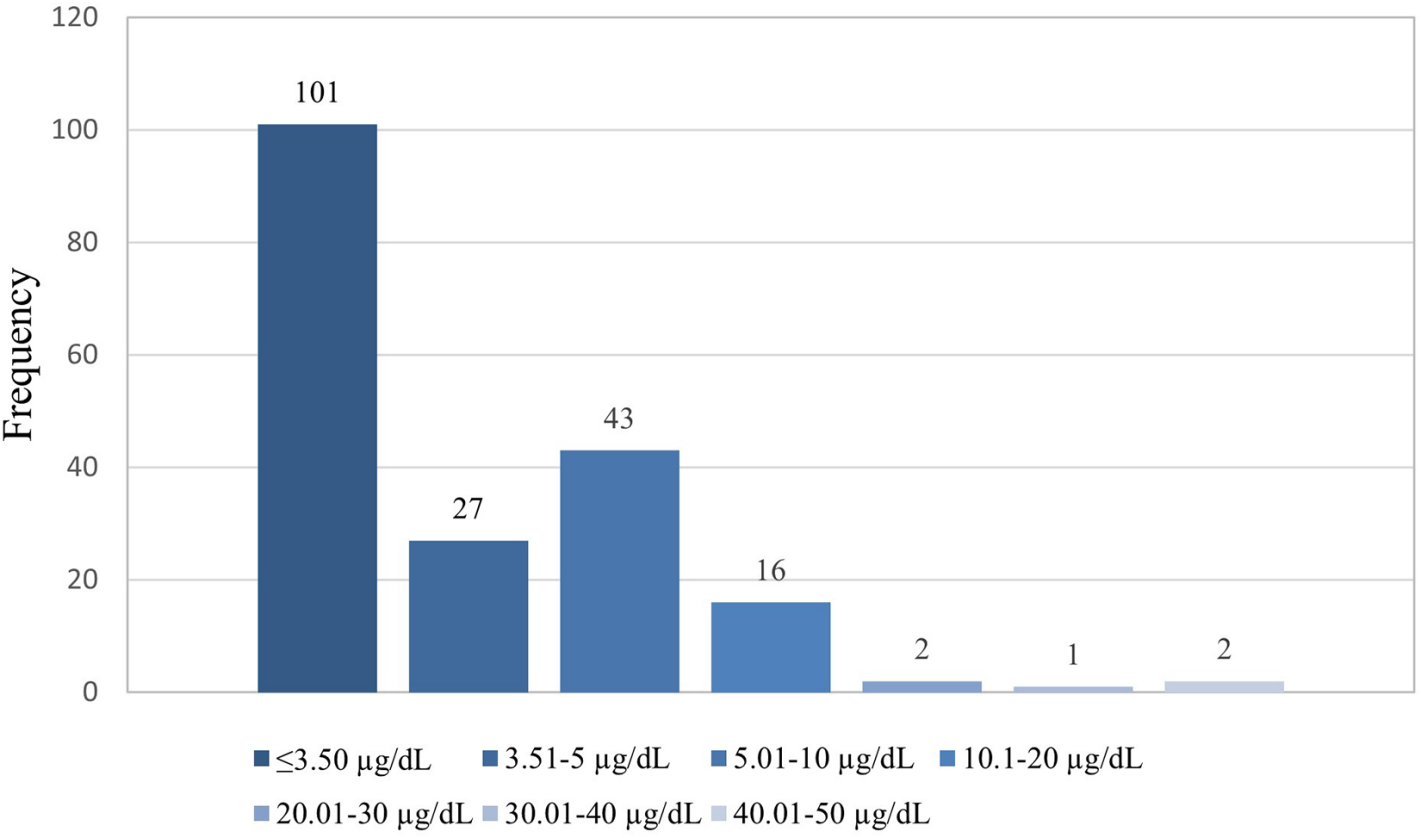
Categorical distribution of blood lead concentration.

The study's overall blood lead concentration median was 3.30 μg/dl and the interquartile (IQR) was 2.00 and 6.45 respectively. The lowest and highest measured blood lead concentrations were 0.85 and 48.11 μg/dl, respectively. The majority of participants in the study had blood lead concentrations of ≤3.50 µg/dl (*n* = 101; 53%), followed by those with blood lead concentrations between 5.01 and 10 µg/dl (*n* = 43; 22%) and the least was found among those with blood lead concentrations between 30.01 and 40 µg/dl (*n* = 1; 1%), as shown in Figure 3.

### 3.2 Socio-demographics of the study participants

Socio-demographics are shown in Table 1 below. Most participants were aged between 16 and 18 years (*n* = 146; 76%), and their blood lead concentration (3.06 μg/dl) was lower than those 15 years or younger who had a higher blood lead median of 3.44 μg/dl. Half of the participants (*n* = 96; 50%) indicated that their highest educational level was high school, and their blood lead concentrations (4.08 μg/dl) were lower than those with no schooling (mean = 9.63 μg/dl). Participants born outside South Africa were few (*n* = 35; 13%) compared to South African-born participants (*n* = 167; 87%), yet their blood lead concentrations were higher (8.78 vs. 2. 87 μg/dl). Participants born in a village or rural area had higher blood lead concentration (4.95 μg/dl) and most (*n* = 22, 24%) had blood lead concentration of ≥3.50 μg/dl. In this study, some participants were working to support their families before coming to the secure facility (*n* = 36; 19%) and blood lead concentrations were higher (5.23 μg/dl) compared to those who were not working (2.88 μg/dl). The participants who did not smoke had a blood lead median of 3.59 μg/dl, while those who were involved in illegal mining had high blood lead concentrations (9.00 μg/dl). Lastly, when comparing blood lead concentration, the analysis showed a statistical difference for educational levels (*p* ≤ 0.01), country of birth (*p* ≤ 0.01), local birth area (*p* = 0.05), working before coming to the facilities and involvement in illegal mining (*p* ≤ 0.01) showed a statistical difference.

**Table 1:** Description of the socio-demographics and blood lead concentration according to socio-demographics.

Characteristics		Total	Median^a^	<3.50 μg/dl	≥3.50 μg/dl	*p*-value^b^
		*N* (%)		*n* (%)	*n* (%)	
Age	15 years or younger	26 (14%)	3.44 μg/dl	13 (13%)	13 (14%)	0.09
	16–18 years	146 (76%)	3.06 μg/dl	82 (81%)	64 (70%)	
	19 years	20 (10%)	4. 32 μg/dl	6 (6%)	14 (15%)	
Educational levels	No schooling	15 (8%)	5.03 μg/dl	1 (1%)	14 (15%)	≤0.01*
	Primary school	81 (42%)	3.21 μg/dl	44 (43%)	37 (41%)	
	High school	53 (28%)	3.43 μg/dl	28 (28%)	25 (28%)	
	Trade school	43 (22)	2.45 μg/dl	28 (28%)	15 (16%)	
Birth country	South Africa	167 (87%)	2. 87 μg/dl	98 (97%)	69 (76%)	≤0.01*
	Outside South Africa	25 (13%)	8.78 μg/dl	3 (3%)	22 (24%)	
Local birth area	Town	30 (16%)	2.54 μg/dl	16 (16%)	14 (15%)	0.05*
	Township	115 (60%)	2.87 μg/dl	68 (67%)	47 (52%)	
	Informal settlement	18 (9%)	3.73 μg/dl	8 (8%)	10 (11%)	
	Village or rural	29 (15%)	4.95 μg/dl	9 (9)	20 (22%)	
Occupational status	Not working	156 (81%)	2.88 μg/dl	92 (91%)	64 (70%)	≤0.01*
	Working	36 (19%)	5.23 μg/dl	9 (9%)	27 (30%)	
Smoking	No	60 (31%)	3.59 μg/dl	30 (30%)	30 (33%)	0.63
	Yes	132 (69%)	3.20 μg/dl	71 (70%)	61 (67%)	
Illegal mining	No	171 (89%)	2.87 μg/dl	171 (100%)	0	≤0.01*
	Yes	21 (11%)	9.00 μg/dl	0	21 (100%)	

^a^ Blood lead concentration median.

^b^ Chi-square test was used to perform statistical significance.

* *p*-value was set at 0.05 for statistical significance.

### 3.3 Environmental and other factors

There were 72 (38%) participants who resided near non-lead emitting industries and had low blood lead concentrations (3.01 µg/dl), which were lower than those who lived near mines or mine tailings (3.74 µg/dl), however, there was no significant difference (*p*-values) when the levels were compared. Few participants who lived in informal housing structures (*n* = 50, 26%) had higher blood lead concentrations (4.44 vs. 2.67 µg/dl) compared to those in formal housing structures (*n* = 142, 74%). Participants who resided in houses where there was no water plumbing (5.22 µg/dl), paint peeling from the inside (3.79 µg/dl) and outside [3.61 µg/dl and most (*n* = 62, 68%) had blood lead concentration above 3.50 µg/dl] had high blood lead concentrations. In addition, participants who lived in a household with a cottage industry in the yard had higher blood lead concentrations (3.80 µg/dl). Lastly, participants living in an extremely overcrowded household and those using traditional medicine (remedies) had elevated blood lead concentrations, with 5.92 and 5.89 μg/dl, respectively. The following environmental factors showed a statistical difference in blood lead concentrations; house structure (*p* = 0.01), plumbing (*p* = 0.01), exterior peeling paint (*p* = 0.03), use of traditional medicine (*p* = 0.01) and cottage business in the yard (*p* = 0.01) The study's environmental risk factors and blood lead concentrations (median) according to environmental factors are described in Table 2.

**Table 2:** Study environmental risk factors.

Risk factors		*n* (%)	Median^a^ (µg/dl)	<3.50 μg/dl	≥3.50 μg/dl	*p*-value^b^
				*n* (%)	*n* (%)	
Residing near lead-emitting sources	Near a mine or mine tailing	72 (38%)	3. 74 µg/dl	34 (47%)	38 (53%)	0.391
	Lead emitting industries	17 (9%)	4.29 µg/dl	8 (47%)	9 (53%)	
	Non-emitting industries	103 (54%)	3. 01 µg/dl	59 (57%)	44 (43%)	
House structure	Formal structure	142 (74%)	2.67 µg/dl	85 (84%)	57 (63%)	0.01*
	Informal structure	50 (26%)	4.44 µg/dl	16 (16%)	34 (37%)	
Plumbing	Metal	136 (71%)	3.29 µg/dl	4 (4%)	16 (18%)	0.01*
	Plastic	36 (19%)	2.36 µg/dl	24 (24%)	12 (13%)	
	No plumbing	20 (10%)	5.22 µg/dl	73 (72%)	63 (69%)	
Exterior peeling paint	No	78 (41%)	2.71 µg/dl	49 (49%)	29 (32%)	0.03*
	Yes	114 (59%)	3.79 µg/dl	52 (51%)	62 (68%)	
Interior peeling paint	No	86 (45%)	2.99 µg/dl	49 (49%)	37 (41%)	0.31
	Yes	106 (55%)	3.61 µg/dl	52 (51%)	54 (59%)	
Use of traditional medicine	No	45 (23%)	2.19 µg/dl	32 (32%)	13 (14%)	0.01*
	Yes	147 (77%)	3.77 µg/dl	69 (68%)	78 (86%)	
Cottage business in the yard	No	54 (28%)	2.57 µg/dl	37 (37%)	17 (19%)	0.01*
	Yes	138 (72%)	3.80 µg/dl	64 (63%)	74 (81%)	
Overcrowding	No overcrowding	17 (9%)	3.93 µg/dl	8 (8%)	9 (10%)	0.80
	Moderate overcrowding	161 (87%)	3.29 µg/dl	88 (87%)	79 (87%)	
	Extreme overcrowding	14 (4%)	4.07 µg/dl	5 (5%)	3 (3%)	

^a^ Blood lead concentration median.

^b^ Chi-square test was used to perform statistical significance.

* *p*-value was set at 0.05 for statistical significance.

### 3.4 Factors that influence blood lead concentrations

The bivariate analysis to show individuals variables associated with high blood lead concentrations among study participants is shown in Table 3 below. Age was not found to be a risk factor for elevated BLLs. The analysis showed that being born outside South Africa (COR: 10.42, 95%CI: 3.00–36.17) and in a village or rural (COR: 2.54, 95%CI: 3.00–36.17), working to support a family (COR: 4.31, 95%CI: 1.90–9.78), involved in illegal mining COR: 28.17, 95%CI: 3.69–4.75), living in a house with plastic plumbing (COR: 1.13, 95%CI: 1.03–1.46) and residing in a house external peeling paint (COR: 2.01, 95%CI: 1.12–3.63) were found to influence blood lead concentrations in the study. The bivariate analysis showed that the following risk factors influenced blood lead concentrations: being a smoker (COR: 4.31, 95%CI: 1.98–9.78), residing in an informal structure (COR: 3.17, 95%CI: 1.60–6.72), using traditional medicine (COR: 3.78, 95%CI: 1.35–5.72), and have a cottage business in the yard (COR: 2.52, 95%CI: 1.29–4.89).

**Table 3:** Bivariate analysis between blood lead concentration with socio-demographics and environmental factors.

Risk factor		Crude odds ratio	*p*-value	95% confidence interval	
				Lower	Upper
Age	≤15 years	Ref			
	16–18 years	0.78	0.56	0.34	1.80
	19 years	2.33	0.18	0.68	7.96
Educational levels	No schooling	Ref			
	Primary	0.06	0.01*	0.01	0.48
	High school	0.06	0.01*	0.01	0.52
	Trade school	0.04	≤0.01*	0.00	0.32
Birth country	South Africa	Ref			
	Outside South Africa	10.42	0.05*	3.00	36.17
Local birthplace	Town/City	Ref			
	Township	0.79	0.57	0.35	1.77
	Informal settlements	1.43	0.55	0.44	4.62
	Village or Rural	2.54	0.09	3.88	7.36
Occupational status	No	Ref			
	Yes	4.31	≤0.01*	1.90	9.78
Smoking	No	Ref			
	Yes	4.31	≤0.01*	1.98	9.78
Illegal mining	No	Ref			
	Yes	28.17	0.02*	3.69	4.75
Residing near lead-emitting sources	Non-lead emitting industries	Ref			
	Lead emitting industries	0.67	0.03*	0.36	1.22
House structure	Formal structure	Ref			
	Informal structure	3.17	≤0.01*	1.60	6.27
Plumbing	No plumbing	Ref			
	Plastic	1.13	≤0.01*	1.03	1.46
	Metal	1.22	0.01*	1.07	1.68
Exterior peeling paint	No	Ref			
	Yes	2.01	0.02*	1.12	3.63
Interior peeling paint	No	Ref			
	Yes	1.38	0.27	0.78	2.44
Use of traditional medicine	No	Ref			
	Yes	3.78	0.01*	1.35	5.72
Cottage business in the yard	No	Ref			
	Yes	2.52	0.01*	1.29	4.89
Overcrowding	No overcrowding	Ref			
	Moderate Overcrowding	0.80	0.66	0.29	2.17
	Extreme Overcrowding	0.53	0.04*	0.10	2.98

* *p* < 0.05 = statistically significant.

The multivariate analysis model (using the backward stepwise elimination method) presented in Table 4 shows risk factors associated with high blood lead concentrations in the study population. Young males who were born outside of South Africa (AOR: 3.10, 95%CI: 1.01–1.88), involved in illegal mining (AOR: 1.36, 95%CI: 1.14–1.91), and residing in a house with peeling paint on the outside (AOR: 2.26, 95%CI: 1.12–4.30) were more likely to have high blood lead concentrations.

**Table 4:** Multivariate analysis showing factors influencing blood lead concentration.

Risk factor		Adjusted odds ratio	*p*-value	95% confidence interval	
				Lower	Upper
Birth country	South Africa	Ref			
	Outside South Africa	3.10	0.04*	1.01	1.88
Illegal mining	No	Ref			
	Yes	1.36	0.03*	1.14	1.91
Exterior peeling paint	No	Ref			
	Yes	2.26	0.01*	1.12	4.30

* *p* < 0.05 = statistically significant.

## 4 Discussion

The study aimed to determine the blood lead concentrations and risk factors of elevated blood lead concentrations among young males in conflict within a low- and middle-income country, especially in Sub-Saharan. The blood median in this current study was 3.30 μg/dl, while the lowest and highest blood lead concentrations were 0.85 and 48.11 μg/dl, respectively. The study recorded low blood lead concentrations compared to other studies among similar populations (27, 36). A study in New Zealand among similar populations reporting childhood blood lead concentrations reported the highest blood lead concentration of 31.00 µg/dl (27). In our study, the highest blood lead concentration recorded was 48.11 μg/dl. This observation may be attributed to the differing environmental factors and the potential for lead exposure, as New Zealand is classified as a high-income country, whereas South Africa is classified as a middle-income country. The study recorded the highest blood lead concentration when compared with previous studies in South Africa (34–36). Yet, the blood level concentrations were lower than those in the previous study with blood lead concentration median conducted in South Africa among a population with violent behaviour issues (36). Blood lead concentrations were high among young males 19 years old (4.32 μg/dl), those that reside near lead emitting industries (4.29 µg/dl), those residing in informal structures (4.44 µg/dl), those using traditional medicine (3.77 µg/dl) and having a cottage industry in the yard (3.80 µg/dl). These sources have been previously identified to influence human lead concentrations in most LMICs, including South Africa and elsewhere (9, 20, 46, 47).

According to Mathee (9), LMICs such as South Africa have made some strides however, these countries still have challenges with contemporary and emerging risk factors for lead poisoning. In our study, we found the following co-existing factors (being born outside the country, involvement in illegal mining activities and staying in a house with peeling paint on the outside) that influenced blood lead concentration among young males in conflict with the law. Previous studies have highlighted that younger children either born in another country or relocated during their early years are prone to high/low blood lead concentrations (48–50). A study in Greece showed that the prevalence of high blood lead concentrations was higher in children born outside (27.1%) of Greece compared to those born (1.2%) in Greece. Similarly in the United States of America, refugee children have been reported to have high blood lead concentrations compared to American-born children (15, 50–52).

Researchers have raised concerns about the activities of informal sectors contributing to high blood lead levels among surrounding communities and those involved with such activities (9, 24, 25). Recently studies have reported emerging factors (such as informal foundries for cookware) for high blood lead concentration, however, there is little information on informal mining activities (24, 25). In the current study, young males involved in illegal mining activities had blood lead concentrations of 9.00 µg/dl. Furthermore, being involved in illegal mining was found to be associated (*p* = 0.03) with high blood lead concentrations. A study in Zamfara state (Nigeria) reported high blood lead concentrations (ranging from 15 to 561.2 μg/dl) due to illegal gold ore processing among illegal miners. Such activities do not only affect those that are involved but it has an adverse impact on surrounding communities and families. In Nigeria, children aged 6 months to 14 years living near artisanal gold mining activities had blood lead concentrations ranging from 13.4 to 35.8 µg/dl (53). While in 2010, a fatal lead poisoning outbreak was reported where 118 children younger than 5 years old had died due to lead exposure (20). Despite this evidence, there are no studies in South Africa that have been conducted in communities residing near areas where illegal mining is prevalent. Based on this finding, we recommend more research among such vulnerable communities in South Africa to protect children from emerging risks and sources such as illegal mining activities as proven elsewhere. Furthermore, these children might live in dilapidated and unattended houses with peeling lead paint. Therefore, the current study highlights the possible link between elevated blood lead concentrations and residing in a house with peeling paint. This supports scientific knowledge indicating that the house condition, especially those with peeling paint or house conditions, is a risk factor for lead poisoning (9, 54).

According to anecdotal evidence, children who are involved in illegal mining activities are forced to stay in abandoned and dilapidated houses that used to belong to the mines. The majority of those involved in illegal mining activities in the current study were born outside and were working for someone as previously reported in a case report (55). Therefore, we believed that the co-existence of the identified risk factors contributed to the high blood lead concentration in the study population. Based on the study findings, we recommend a multi-sectoral approach to addressing the phenomenon of lead exposure via illegal mining activities and early screening intervention programmes. Furthermore, there should be more research on those involved in illegal mining and surrounding communities.

The study's strength is that blood lead concentrations were analysed in an accredited laboratory, a method deemed superior for lead detection in humans. Another strength, according to the best knowledge of the researcher, this is the first study in the sub-Saharan region to describe blood lead concentrations among the population in conflict with the law (a vulnerable population group). The results of this study can be used as a baseline for future studies and public health action in South Africa and other similar countries. The limitations of the study were that a cross-sectional study design was used. Consequently, blood lead concentrations were measured at one point, and only current (recent) blood lead concentrations were measured in the present study. Hence, we recommended that lifetime lead concentrations or blood lead collected at different intervals be used, as lead concentrations may vary or increase over time. Secondly, the study primarily concentrated on delineating the prevalence and risk factors associated with blood lead concentration. Consequently, we recommend conducting cohort or case-control studies to compare these findings with those of young males who are not in conflict with the law. Such research would facilitate the identification and understanding of additional risk factors associated with behavioural issues among young males in conflict with the law. Lastly, the study participants were conveniently sampled, which could have led to selection bias, but it was managed throughout.

## 5 Conclusion

The study reported a prevalence of high blood lead concentration among young males in conflict with the law in an LMIC. It also highlights the influence of co-existing (contemporary) factors on human lead concentrations, especially blood. Furthermore, identified the emergence of illegal mining activities as a risk factor for high blood lead concentrations. Therefore, screening for blood lead in vulnerable populations affected by societal issues is essential. Further research is needed to examine the lifetime human lead concentrations and other risk factors among young males in conflict with the law in a large sample and other population correctional facilities (prison).

## Data Availability

The datasets presented in this study can be found in online repositories. The names of the repository/repositories and accession number(s) can be found in the article/Supplementary Material.
